# Antifungal Activity of Isolated *Bacillus amyloliquefaciens* SYBC H47 for the Biocontrol of Peach Gummosis

**DOI:** 10.1371/journal.pone.0162125

**Published:** 2016-09-01

**Authors:** Xunhang Li, Yanzhou Zhang, Zhiwen Wei, Zhengbing Guan, Yujie Cai, Xiangru Liao

**Affiliations:** 1 The Key Laboratory of Industrial Biotechnology, Ministry of Education, School of Biotechnology, Jiangnan University, 1800 Lihu Avenue, Wuxi, 214122, China; 2 The Bioscience and Engineering College, Jiangxi Agriculture University, Nanchang, 330045, China; National Renewable Energy Laboratory, UNITED STATES

## Abstract

The gummosis disease is caused by *Botryosphaeria dothidea* (Moug. ex. Fr) Ces. et de Not., and it is one of the most important diseases of stone fruits worldwide. The use of biocontrol as an alternative approach to synthetic chemical fungicides has aroused general concern about how to control plant diseases that are caused by phytopathogens. The aim of this study is to isolate *Bacillus* strains from raw honeys with the capacity to inhibit *B*. *dothidea* and to explore the mechanisms by which they could be used in the biocontrol of peach gummosis. *Bacillus amyloliquefaciens* SYBC H47 was isolated and identified on the basis of its physiological and biochemical characteristics and its 16S rRNA and *gyrB* gene sequences. The cell suspension and the cell-free supernatant of its culture showed significant antifungal activity against *Aspergillus niger*, *Mucor racemosus*, *Fusarium oxysporum*, *Penicillium citrinum*, and *Candida albicans* by agar-diffusion assays. The primary antifungal substances were bacillomycin L, fengycin, and surfactin, which were analyzed by HPLC LC/ESI-MS/MS. Bacillomycin L showed the best inhibitory effect against conidial germination of *B*. *dothidea*, followed by fengycin and surfactin. Surfactin had limited effects on mycelial growth, contrary to those of bacillomycin L and fengycin. However, a mixture of the three lipopeptides had a synergistic effect that disrupted the structure of the conidia and mycelia. In order to reduce the production cost, the use of waste frying peanut oil and soy oil as the sole carbon source increased the lipopeptide yield levels by approximately 17% (2.42 g/L) and 110% (4.35 g/L), respectively. In a field trial, the decreases in the infected gummosis rate (IGR) and the disease severity index (DSI) through cell suspension treatments were 20% and 57.5% (in 2014), respectively, and 40% and 57.5% (in 2015), respectively, in comparison with the control. In conclusion, *B*. *amyloliquefaciens* SYBC H47 could inhibit the germination of conidia and the growth of mycelia from *B*. *dothidea*; therefore, this strain behaves as a potential biocontrol agent against the gummosis disease.

## Introduction

The peach is an economically important fruit in China. However, peach gummosis leads to gum exudation on trunks, scaffold limbs, and branches, and it significantly depresses the tree growth and fruit yield of susceptible peach varieties [1]. Previous studies suggested that *Botryosphaeria* species are pathogenic fungal species that cause gummosis in peaches and other stone fruits, such as apricots, almonds, and cherries [1]. Among them, *Botryosphaeria dothidea* (Moug. ex. Fr) Ces. et de Not., an ascomycete fungus, is the dominant contagious pathogenic fungus species that appears during the peach production period [2]. The conidia of *B*. *dothidea* invade peach trees leading to latent infection via pruning wounds, damaged tissue, lenticels, and dormant buds [3]. In addition, the overwintering *B*. *dothidea*, which is embedded in the bark and branches of diseased trees, is the primary inoculum that provides conidia for the next few years [4].

To date, chemical synthetic fungicides, namely, carbendazim, thiophanate methyl, captafol, and captan, have been used to control gummosis diseases in orchards [1, 5]. However, their control effects were very limited. The reasons for this limitation may consist of the following: (1) the conidial germination and mycelia growth occur within the bark of trees, where chemical fungicides cannot permeate [4]; and (2) long-term chemical synthetic fungicide abuse has induced fungicidal resistant in pathogens [6]. In view of the need to reduce environmental risks and satisfy health concerns, biocontrol is viewed as an alternative treatment that can replace the use of synthetic chemical fungicides.

*Bacillus* species have the ability to form endospores and synthesize a vast amount of metabolites, with the exception of toxin-producing *Bacillus anthracis* and *Bacillus cereus*, and they are often considered beneficial and safe to plants and the ecological environment [7]. These properties of *Bacillus* species make them good biocontrol agents for substituting synthetic chemical fungicides. *Bacillus* species, with *Bacillus subtilis* and *Bacillus amyloliquefaciens* in particular, play a prominent role in protecting plants from pathogens and promoting plant growth in terms of their capacity to colonize plant roots [8–10]. Several studies show that antimicrobial substances that are biosynthesized by *Bacillus* species are bacteriocins (which are synthesized ribosomally) and lipopeptides (synthesized non-ribosomally) [11, 12]. Furthermore, lipopeptides have attracted more attention because they have many desirable characteristics, such as strong antimicrobial activities, broad antimicrobial spectrum, antiviral and antitumor activities, low toxicity, high temperature tolerance, and high biodegradability [13]. With respect to the biocontrol of plant diseases, the most-studied lipopeptides are the iturin, fengycin and surfactin families for their antagonistic action against a variety of fungal phytopathogens, such as *Rhizoctonia solani* [14], *Pythium ultimum*, *Botrytis cinerea* [15], *Podosphaera fusca* [16], *Fusarium graminearum* [17, 18], and *Sclerotinia sclerotiorum* [19]. Although many studies have reported on the antimicrobial substances that are produced by *Bacillus* species for the biocontrol of different plant diseases, few investigations about the biocontrol of peach gummosis as caused by *B*. *dothidea* have been reported.

In comparison with synthetic chemical fungicides, the commercialization of lipopeptides by industry is limited by lower yields and higher production costs. According to estimates, substrates account for 10–30% of the total production costs associated with the fermentation processes. Thus, using low-cost raw substrates will reduce the production cost. Several studies have reported that various plant oils and waste oils are suitable for microbial growth and can be used as effective and cheap substrates for lipopeptide production, such as sunflower oil, soybean oil, corn oil and rapeseed oil [20]. Furthermore, recent investigations have focused on reducing rhamnolipid, sophorolipid, and glycolipid production costs by using various plant oils as raw substrates [21–23]. Few investigations have addressed lipopeptides that are synthesized by *Bacillus* species.

The objective of the present study was to isolate *Bacillus amyloliquefaciens* SYBC H47 from raw honeys; this strain had the capacity to effectively suppress *B*. *dothidea* (Moug. ex. Fr) Ces. et de Not. and was identified on the basis of physiological and biochemical properties tests and 16S rRNA and *gyrB* gene sequence analyses. Antimicrobial substances were extracted, purified and identified. The effects of conidial germination, mycelial growth and cell membrane permeability were evaluated *in vitro*. Low-cost raw substrates were evaluated for their lipopeptide yields. The effects of the biocontrol were evaluated in field trials.

## Materials and Methods

### Isolation of bacterial strains

The method of bacterial strain isolation was used as described by Lee *et al* [24] with minimal modifications. Honey solution (50% w/v) was made by diluting 1 g of raw honey (from eucalyptus in Fuyuan, Guangxi province, China) in 1 mL of sterile water. A 100 μL volume of different raw honey solutions (50% w/v) was spread on Luria Bertani Agar (LBA) plates, which contained (g/L) tryptone, 10; yeast extract, 5; NaCl, 10; and agar, 18, at pH 7.0, and the solutions were then incubated at 30°C for 48 h. The isolates were streaked on fresh LBA plates and stored at 4°C for further study.

### Antifungal activity test *in vitro* against *B*. *dothidea*

The antifungal activity was detected by performing confrontation assays against *B*. *dothidea* in dual culture plates. In brief, the bacterial isolates were streaked onto potato dextrose agar (PDA) plates containing (g/L) potato, 200; glucose, 20; and agar, 18, by using sterile toothpicks to draw thin lines at a 22 mm distance from the center. Opposite side, placed a 5 mm-diameter mycelial plug chopped by a sterile cork borer from the edge zone of the *B*. *dothidea* PDA plate which was grown at 25°C for 6 days. The plates were incubated at 28°C until the inhibition zone of *B*. *dothidea* was formed. All these tests were repeated in triplicate.

The percentage growth inhibition rate (GIR; %) was expressed as described by [25].

GIR(%)=[(C−T)/C]×100

where C is the mycelial growth of the control (mm), and T is the mycelial growth in treatment (mm).

### Bacterial strain identification

Phenotypic characterizations were conducted as described by Bergey [26], and carbon source utilization was tested with API 50 CH carbohydrate fermentation strips according to the manufacturer’s instructions (bioMérieux, Inc.) [27]. A whole cell fatty acid analysis was performed as described by Sharma [28].

### 16S rRNA gene and *gyr*B gene sequencing and phylogenetic analysis

To extract genomic DNA, the isolate was cultured overnight in LB medium. Bacterial cells were collected by centrifugation at 6,790 *g* for 10 min. Genomic DNA was extracted by using a DNA Mini-Prep kit (Sangon Biotech Co., Ltd., China) according to the manufacturer’s instructions. A fragment of the 16S rRNA gene was amplified by PCR using the universal primer pair 27-F (5'-AGA GTT TGA TCC TGG CTC AG-3') and 1492-R (5'-GGT TAC CTT GTT ACG ACT T-3') [29]. A fragment of the *gyrB* gene was amplified by PCR using UP-1-F (5'-GAA GTC ATC ATG ACC GTT CTG CA -3') and UP-2-R (5'-AGC AGG GTA CGG ATG TGC GAG CC -3') [30]. The PCR products were then sequenced by Sangon Biotech Co., Ltd., China. Phylogenetic trees were constructed using MEGA 4 following the multiple alignment of the sequences by Clustal X 2.0. The trees were constructed using the Neighbor-Joining (NJ) method and the maximum parsimony options model, with 1000 bootstrap replicates to estimate the support for each branch.

### Production of hydrolytic enzymes and plant growth-promotional compounds

Protease activity detection was performed by measuring the clearing zone around each colony on LB plates amended with 2% skim milk powder after incubation for 48 h at 30°C as described by [31]. The chitinase activity was assayed on chitin minimal agar medium by adding 0.2% colloidal chitin [32]. The β-1,3-glucanase activity was examined on minimal medium (MM) by adding laminarin (β-1,3-glucan) as the sole carbon source [33]. In brief, the isolate was inoculated onto the center of the plates and cultured at 30°C for 7 days, and the clearing zones around the colonies were observed. The production of indoleacetic acid (IAA) by the isolate was examined as described by [34]. The siderophore production of the isolate was tested as described by [35]. The phosphate solubilization was detected on Pikovskaya’s agar medium containing tricalcium phosphate as described by [36].

### Antifungal spectrum of the isolate

The isolate was cultured in 250-mL Erlenmeyer flasks containing 30 mL of LB medium at 30°C for 48 h with shaking at 200 rpm. The cell-free supernatant of the isolate was centrifuged at 12,000 *g* for 20 min to remove the bacterial cells. The antifungal activities of the cell suspension and the cell-free supernatant were evaluated in relation to their ability to suppress several pathogens, including *B*. *dothidea*, *A*. *niger*, *M*. *racemosus*, *F*. *oxysporum*, *P*. *citrinum*, and *C*. *albicans*. Uninoculated LB medium was used as a negative control. The fungal pathogenic strains were used as indicator fungi, and they were incubated on PDA plates at 28°C for 5 days. A 5 mm-diameter mycelial plug of indicator fungi was placed at a 22 mm distance from the centers of the PDA plates. An Oxford cup was placed on the opposite side and a 100 μL sample was injected into the Oxford cup by manual liquid handling. The plates were incubated at 30°C, until an inhibition zone was formed.

### Isolation of the antifungal substances

The antifungal substances were isolated by following the method described in [16]. In brief, the supernatant of the isolate was adjusted by adding 6 mol/L HCl until a pH of 2 was reached, and then it was preserved overnight at 4°C. The precipitate was collected by centrifugation (10,000 *g* for 20 min), then resuspended in distilled water and adjusted to pH 7.0 with 2 mol/L NaOH. After it was freeze-dried, the resulting brown freeze-dried substance was extracted three times with methanol, and then the extracting methanol solutions were merged and vacuum-dried. The extract was resuspended in 1 mL of methanol.

### HPLC LC/ESI-MS/MS

For API-ESI analysis, 1 μL of purified antifungal substances was loaded onto an HPLC (Waters Acquity UPLC, Waters Corporation, Maple Street Milford, MA, USA) that had a Waters MALDI SYNAPT Q-TOF MS mass spectrometer equipped with an ESI source. Separations were performed on an ACQUITY UPLC BEH C_18_ RP column (2.1 × 50 mm, 130 Å, 1.7 μm) and eluted at a flow rate of 0.3 mL/min, and they were monitored with a Waters ACQUITY photodiode array (PDA) detector at 210 nm. Mobile phase A consisted of 0.1% formic acid in methanol, and phase B consisted of 0.1% formic acid in Milli-Q water. The gradient conditions were 0–3 min, 15% A; 3–15 min, 40–100% A; 17 min, 100% A; 17.1 min, 15% A; and 20 min, 15% A.

All the peaks separated by the HPLC system were infused into the MALDI SYNAPT Q-TOF MS and fractionated for further analysis. Ions were acquired in positive-ion mode using electrospray ionization mass spectrometry (ESI-MS) at a capillary voltage of 3.5 kVolts and a sample cone voltage of 30 Volts. The source block and desolvation temperatures were set to 100°C and 400°C, respectively. The scan ranged from *m/z* 50 to 2,000.

### Thin layer chromatography (TLC)

Antifungal substances were purified by TLC as described in [37]. The crude extract was dissolved in methanol and analyzed by thin layer chromatography (TLC) on silica gel 60 plates (F254), with a solvent system consisting of chloroform-methanol-water (65:15:4, v/v). After the separation, the spots were revealed with iodine vapor. Color fractions were scraped and collected, and they were redissolved in deionized water. Antifungal substances were obtained from different water solutions by lyophilization.

### Effects of the lipopeptides on the suppression of conidial germination

*B*. *dothidea* was inoculated on a PDA plate and incubated for 7 days at 28°C. The fungal conidia were harvested by adding sterile water and stirring with a sterile glass rod and then subsequently filtering the liquid to remove the mycelia with sterile degreasing cotton. One milliliter of conidia (10^4^/mL) was added to polypropylene microcentrifuge tubes containing different concentrations (0, 50, 100, 200, and 300 μg/mL) and different types of lipopeptides for 2 h. The lipopeptide mixture was made of bacillomycin L: fengycin: surfactin (1:1:1, v/v/v). The microcentrifuge tubes were centrifuged at 10,000 rpm for 15 min, washed twice with sterile water, and then resuspended in PDB at 28°C for 4 h. The optical microscope was used to examine and calculate the conidial germination rates. The suppression of the conidial germination rate (SCGR; %) was calculated using the following formula:

SCGR(%)=(1−Nt/Nc)×100

where N_t_ is numbers of germinated conidia in the treatment, and N_c_ is number of germinated conidian in the control.

### Effects of lipopeptides on the suppression of mycelial growth

Agar diffusion assays were used to assess the antifungal properties of different lipopeptides as described in the antifungal spectrum of antifungal substances. The concentration of the bacillomycin L, fengycin, surfactin and lipopeptides mixtures was 100 μg/mL.

### Effects of lipopeptides on cell membrane permeability

The cell membrane permeability was examined according to [38] with minor modifications. In brief, five mycelial plugs (5 mm diameter) that were cut from the margin of a 5-day-old colony on PDA were transferred into 50 μg/mL lipopeptide-amended PDB at 50 mL/250 mL flask. A no-lipopeptide liquor was used as the control. The flasks were cultured at 28°C and 180 rpm for 3 days. The mycelia were collected by vacuum filter, washed twice with distilled water, and vacuum-filtered for 15 min. The mycelia (0.5 g) were resuspended in 20 mL of distilled water. The cell membrane permeability was expressed as the electrical conductivity of the distilled water at the interval times (0, 10, 20, 30, 40, 50, 60, 90, 120, 150, and 180 min) using a conductivity meter (JENCO VISIONPLUS 3175, China). The mycelial liquor was boiled for 5 min, and the final electrical conductivity was determined after equilibration at 28°C. The relative conductivity of the mycelia was calculated using the following formula:

relative conductivity(%)=(conductivity/final conductivity)×100.

### Low-cost carbon source and lipopeptide production medium

Landy medium [39] was used as a basal medium to produce lipopeptides by submerged fermentation. Different oil substrates (with 2% concentration substitutes for glucose) such as frying peanut oil, frying soy oil, and frying palm oil were tested for lipopeptide production by strain SYBC H47. The frying oils were collected from a local bakery and confectionery and filtered through muslin cloth to remove the solids. The inoculum volume (5%) of the bacterial cell suspension that was prepared from an 8-h culture in LB medium was placed in 250-mL Erlenmeyer flasks containing 40 mL of pre-sterilized fermentation medium and then incubated at 200 rpm and 30°C for 48 h. The production of lipopeptides was assayed by HPLC.

### Field trials

The efficacy of the isolate for use in the biocontrol of gummosis was evaluated in orchards using the even-aged peach trees at Wuxi (Jiangsu, China) in May 2014 and April 2015. These trees were 7 years old, and they had been seriously infected by *B*. *dothidea* for several years. Cell suspensions were prepared as follows: bacterial cells were collected by centrifugation at 6,790 g for 10 min and then diluted with lipopeptide liquor (100 μg/mL) at 2×10^6^ cfu/mL. Four treatments, namely the cell suspension, lipopeptide liquor (100 μg/mL), carbendazol (200 μg/mL) and water, were sprayed on the trunks to suppress gummosis disease. Each treatment had 10 replications, and gummosis disease was observed and analyzed after 30 days. The calculation of the infected gummosis rate (IGR; %) and the disease severity index (DSI; %) were described in [40]. The IGR was calculated with the following formula:

IGR(%)=(symptomatic plants/total plants)×100;

and the DSI was calculated with the following formula:

DSI(%)=[∑(rating of each plant/4)×number of plants rated]×100.

The disease severity was recorded using grades of 0–4, where 0 = no disease; 1 = 1–9 gums; 2 = 10–19 gums; 3 = 20–29 gums; and 4 = above 29 gums.

### Statistical analysis

The SPSS 18.0 software package was used to analyze the significant differences between different treatments and control by one-way ANOVA and Duncan’s test (*P*<0.05).

## Results

### Isolation of antagonism from raw honey

To screen for the strain that had antifungal activity against *B*. *dothidea*, a total of 225 strains that were isolated from raw honey were tested by dual culture technique. Twenty-three strains (10.22%) exhibited antifungal activity against *B*. *dothidea*, among which strain SYBC H47 had the most efficient antagonism and was selected for further study.

### Identification of the antagonistic strain SYBC H47

The colonies of this strain are creamy white and slimy with a ridged surface and skin-like pellicles on LBA. The cells are gram-positive, motile rod-shaped, endospore-forming, and strictly aerobic. Growth occurs at 15–45°C. This strain does not grow at NaCl concentrations above 15% (w/v). It is positive for hemolytic activity. Its citrate utilization, Tween 20, Tween 40, Tween 60, and Tween 80 hydrolysis are variable. Triton X-100 is harmful to its growth. Its Voges-Proskauer test, catalase, and nitrate reduction to nitrite results are positive. Acid is produced from glycerol, L-arabinose, D-ribose, D-xylose, D-glucose, fructose, D-mannose, lactose, sorbitol, inositol, D-mannitol, melibiose, methyl α-D-glucoside, amygdalin, aesculin, cellobiose, maltose, sucrose, trehalose, and starch. However, nitrate reduction to N_2_, L-rhamnose, N-acetylglucosamine, raffinose, potassium gluconate, propionate, fucose, and potassium 2-ketogluconate are negative (Table 1). The major fatty acids in this strain are iso-C_14:0_ (1.40%), C_14:0_ (1.49%), iso-C_15:0_ (39.36%), anteiso-C_15:0_ (0.30%), iso-C_16:0_ (7.84%), C_16:0_ (22.58%), iso-C_17:0_ (13.21%), and C_17:0_ (4.56%). It is resistant to ampicillin up to 200 μg/mL.

**Table 1:** Phenotypic characteristics that differentiate strain SYBC H47 from phylogenetically related *Bacillus* species.

Characteristic	1	2	3	4
Pigmentation	Creamy white	Creamy white	Opaque	Creamy white
NaCl tolerance	15%	10%	10%	4%
Growth temperature (°C)	15–45	15–50	15–55	20–45
Hemolytic activity	+	+	-	+
Acid production from:				
D-Xylose	+	-	+	-
L-Rhamnose	-	+	-	+
N-Acetylglucosamine	-	-	-	+
Lactose	+	+	-	+
Melibiose	+	+	-	+
Raffinose	-	+	+	-
Potassium gluconate	-	-	-	+
Citrate utilization	+	+	-	-
Indole production	+	+	-	-
DNA G+C content (mol%)	46	46	45	45

Taxa: 1, strain SYBC H47 (data from this study); 2, *B*. *amyloliquefaciens* FZB42 [41]; 3, *B*. *subtilis* subsp. *subtilis* 168 (data from this study). 4, *B*. *velezensis* CBMB205 = *B*. *methylotrophicus* KACC 13105 [42]. All the isolates were positive for motility, catalase, strict aerobic growth, nitrate reduction to nitrite and the Voges-Proskauer test. All the taxa were positive for glycerol, L-arabinose, D-ribose, D-glucose, fructose, D-mannose, sorbitol, inositol, D-mannitol, methyl α-D-glucoside, amygdalin, aesculin, cellobiose, maltose, sucrose, trehalose, and starch. All the taxa were negative for nitrate reduction to N_2_, propionate, fucose, and potassium 2-ketogluconate. +, positive; -, negative.

**Fig 1:**
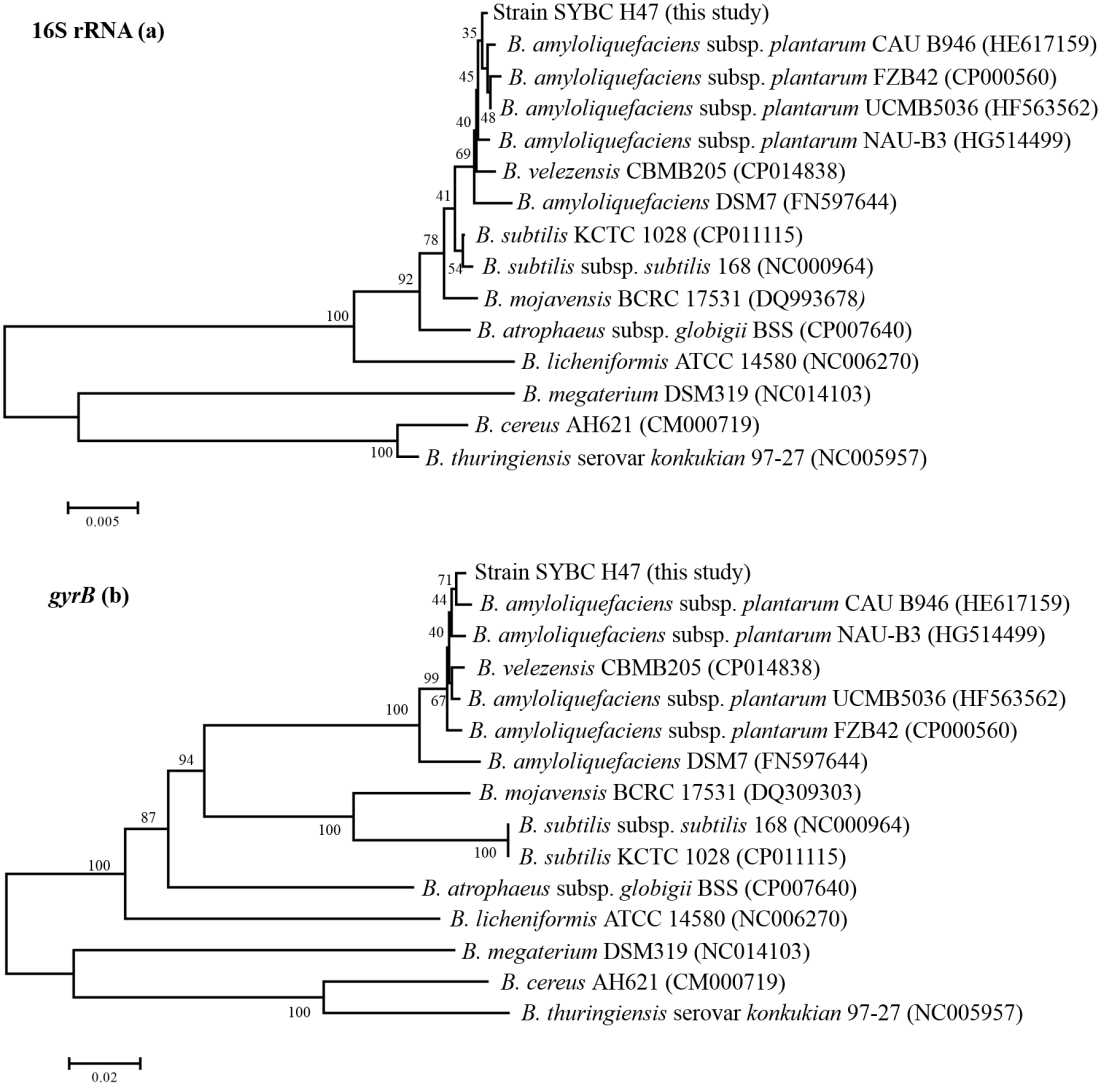
Phylogenetic trees based on 16S rRNA (a) and *gyrB* (b) gene sequences of strain SYBC H47 from bacteria related to *Bacillus* species. The trees were constructed with the neighbor-joining method, and the genetic distances were computed by the maximum parsimony options model. The numbers at the branches denote the bootstrap percentages for 1,000 replicates. Bar, the number of substitutions at certain nucleotide positions.

The phylogenetic tree of the 16S rRNA gene sequences revealed that strain SYBC H47 together with the type strains of *B*. *amyloliquefaciens*, *Bacillus velezensis*, and *B*. *subtilis* formed one cluster, which could be distinguished from another cluster formed by *Bacillus mojavensis*, *Bacillus atrophaeus*, *Bacillus licheniformis*, *Bacillus megaterium*, *Bacillus cereus*, and *Bacillus thuringiensis* (Fig 1a). Strain SYBC H47 shared 99.7–99.9% similarity with *B*. *amyloliquefaciens*, 99.8% similarity with *Bacillus velezensis*, and 99.6% similarity with *B*. *subtilis*. Moreover, strain SYBC H47 shared 93.5–99.4% similarity with other strains according to its 16S rRNA gene sequences (Table 2). Therefore, it is not sufficient to distinguish one species from another according to 16S rRNA gene sequences [43]. The gyrase subunit B (*gyrB*) gene sequence analysis is a new method for phylogenetic classification [30, 44]. Comparatively, strain SYBC H47 shared 99.4% similarity with *B*. *amyloliquefaciens* subsp. *plantarum* CAU B946 and formed one cluster, and it showed 95.9–99.2% similarity with other *B*. *amyloliquefaciens* strains (Fig 1b). However, other members of the related cluster were between 70.9–83%, except that *B*. *velezensis* CBMB205 was 99%.

**Table 2:** Comparison of *gyrB* and 16S rRNA gene sequence similarity between *Bacillus* strains.

*Bacillus* strains	*gyrB*/16S rRNA gene sequence similarity (%)
strain SYBC H47	
*B*. *amyloliquefaciens* subsp. *plantarum* CAU B946	99.4 / 99.9
*B*. *amyloliquefaciens* subsp. *plantarum* UCMB5036	99.2 / 99.9
*B*. *amyloliquefaciens* subsp. *plantarum* NAU-B3	99.0 / 99.9
*B*. *amyloliquefaciens* subsp. *plantarum* FZB42	98.9 / 99.9
*B*. *amyloliquefaciens* DSM7	95.9 / 99.7
*B*. *velezensis* CBMB205	99.0 / 99.8
*B*. *subtilis* KCTC 1028	81.0 / 99.7
*B*. *subtilis* subsp. *subtilis* 168	81.0 / 99.6
*B*. *mojavensis* BCRC 17531	82.0 / 99.4
*B*. *atrophaeus* subsp. *globigii* BSS	83.0 / 99.4
*B*. *licheniformis* ATCC 14580	78.0 / 97.9
*B*. *megaterium* DSM319	72.4 / 93.5
*B*. *thuringiensis* serovar *konkukian* 97–27	71.3 / 93.8
*B*. *cereus* AH621	70.9 / 93.6

Therefore, according to its morphological, physiological, and biochemical characteristics, and the taxonomic analysis of its 16S rRNA and *gyrB* gene sequences, the SYBC H47 strain was identified as *B*. *amyloliquefaciens*. The 16S rRNA and *gyr*B gene sequences were deposited into GenBank under the accession numbers KF863845 and KP271520, respectively. *B*. *amyloliquefaciens* SYBC H47 was preserved at the China Center for Type Culture Collection (CCTCC NO: M2013283), and patent protection was obtained (CN103451128A).

### Enzymatic production and plant growth-promotional attributes

Numerous studies showed that the *Bacillus* species may have different mechanisms when used as biocontrol agents to control disease and promote plant growth. Antifungal enzymes, including chitinase, β-1,3-glucanase, and protease, play important roles in the growth suppression of phytopathogenic fungi. *B*. *amyloliquefaciens* SYBC H47 could form a clearing zone by hydrolyzing skim milk and chitin, and it grows on medium containing laminarin azure, which indicated that it was positive for protease, chitinase and β-1,3-glucanase. It is well-known that IAA, siderophore and phosphate solubilization can promote plant growth. The production levels of IAA and siderophores in *B*. *amyloliquefaciens* SYBC H47 were positive. However, the phosphate solubilization activity was negative.

### Antifungal spectrum activity assay

**Fig 2:**
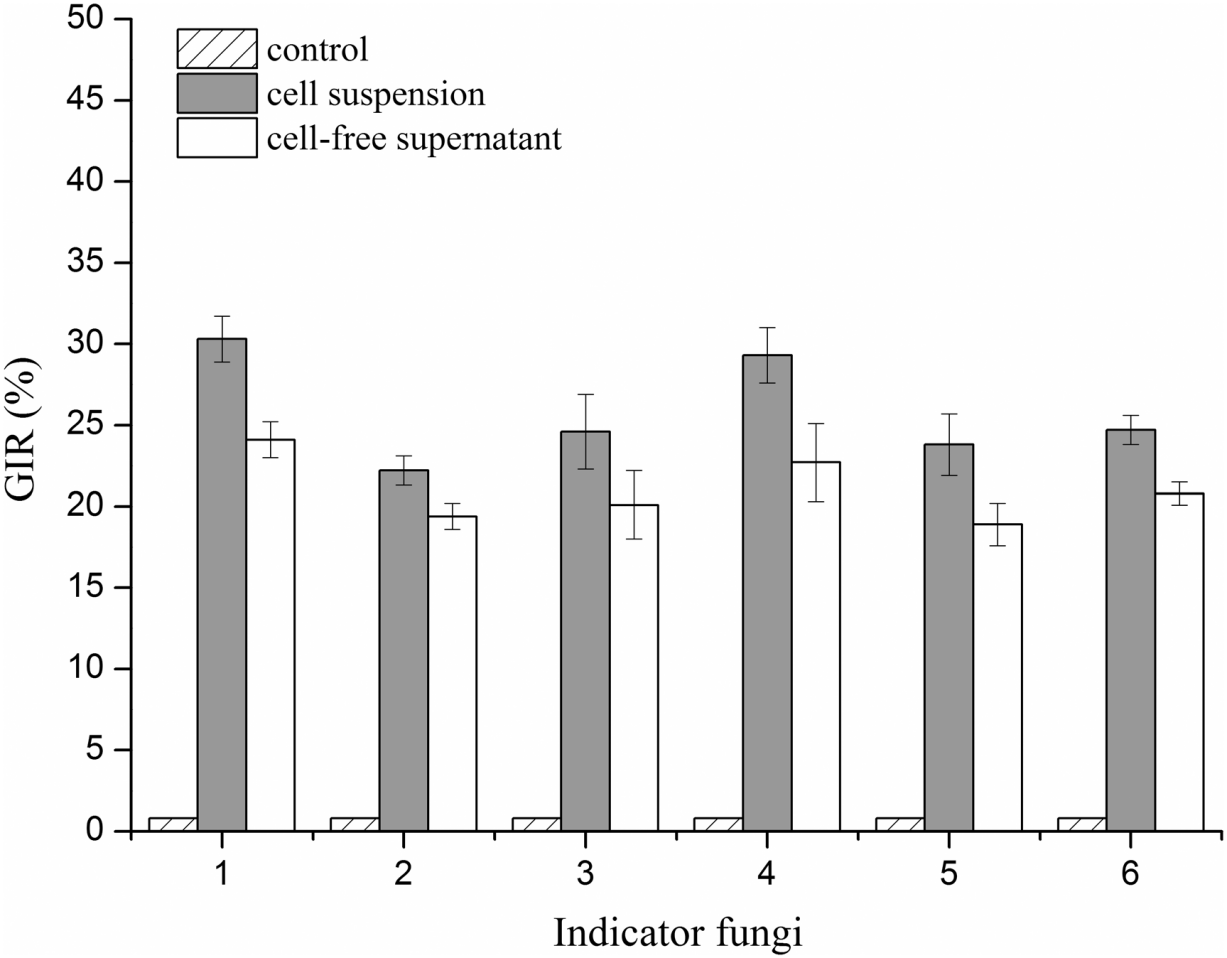
GIR of cell suspension and cell-free supernatant against six indicator fungi. 1: *B*. *dothidea*; 2: *A*. *niger*; 3: *M*. *racemosus*; 4: *F*. *oxysporum*; 5: *P*. *citrinum*; and 6: *C*. *albicans*. Gray columns represent cell suspension, and white columns represent cell-free supernatant.

The antifungal activities of the cell suspension and cell-free supernatant of the SYBC H47 culture were evaluated using the Oxford cup diffusion method. As shown in Fig 2, both the cell suspension and cell-free supernatant exhibited significant antifungal activity against indicator fungi during a PDA *in vitro* bioassay in comparison with that of the negative control (water). In addition, the GIR results of the cell suspension were higher than that of the cell-free supernatant, and they were increased by 2–6%. The maximum GIR of the cell suspension was observed towards *B*. *dothidea* (approximately 30%), and the minimum GIR was found for *A*. *niger* (approximately 22%). These suspensions also exhibited significant GIR levels against the other four pathogenic fungi, namely *F*. *oxysporum*, *C*. *albicans*, *P*. *citrinum*, and *M*. *racemosus*.

### Identification of antifungal substances by HPLC LC/ESI-MS/MS

**Fig 3:**
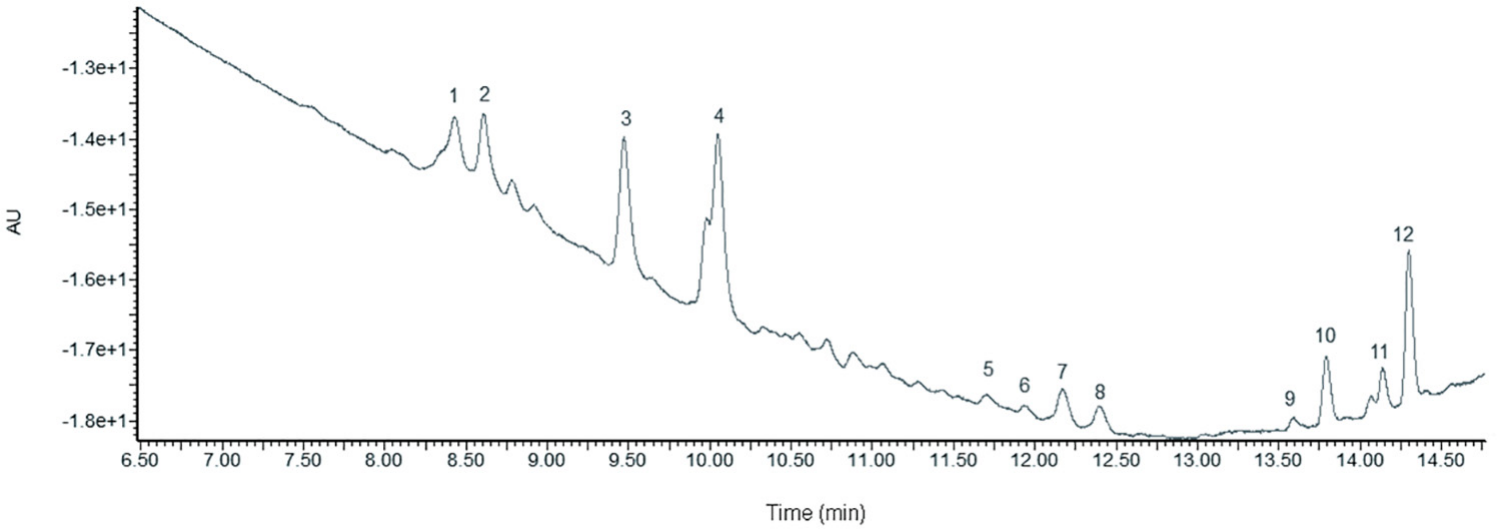
RP-HPLC chromatogram of antifungal substances at 210 nm. The peaks with numerical labels were infused into the MALDI SYNAPT Q-TOF MS and fractionated for further analysis.

For the purpose of this investigation, the substances responsible for antifungal activity and the methanolic fractions from the acid precipitation of cell-free supernatant from the SYBC H47 strain were analyzed by HPLC LC/ESI-MS/MS. The lipopeptide is composed of peptides and β-hydroxy fatty acid with different chain-length carbon atoms. The sufficient separation of lipopeptide isoforms was obtained by RP-HPLC (Fig 3). The retention times ranged from 8.00–10.50 min was correspond to iturin families; 11.50–12.00 min was attributed to fengycin families; and 13.00–14.50 min was associated with surfactin families. There were twelve major fractions that were marked as fractions 1 to 12 and analyzed by MALDI-TOF-MS, and the retention times of the elution were 8.43, 8.62, 9.48, 10.07, 11.66, 11.90, 12.13, 12.35, 13.53, 13.80, 14.11, and 14.31 min, accordingly.

**Fig 4:**
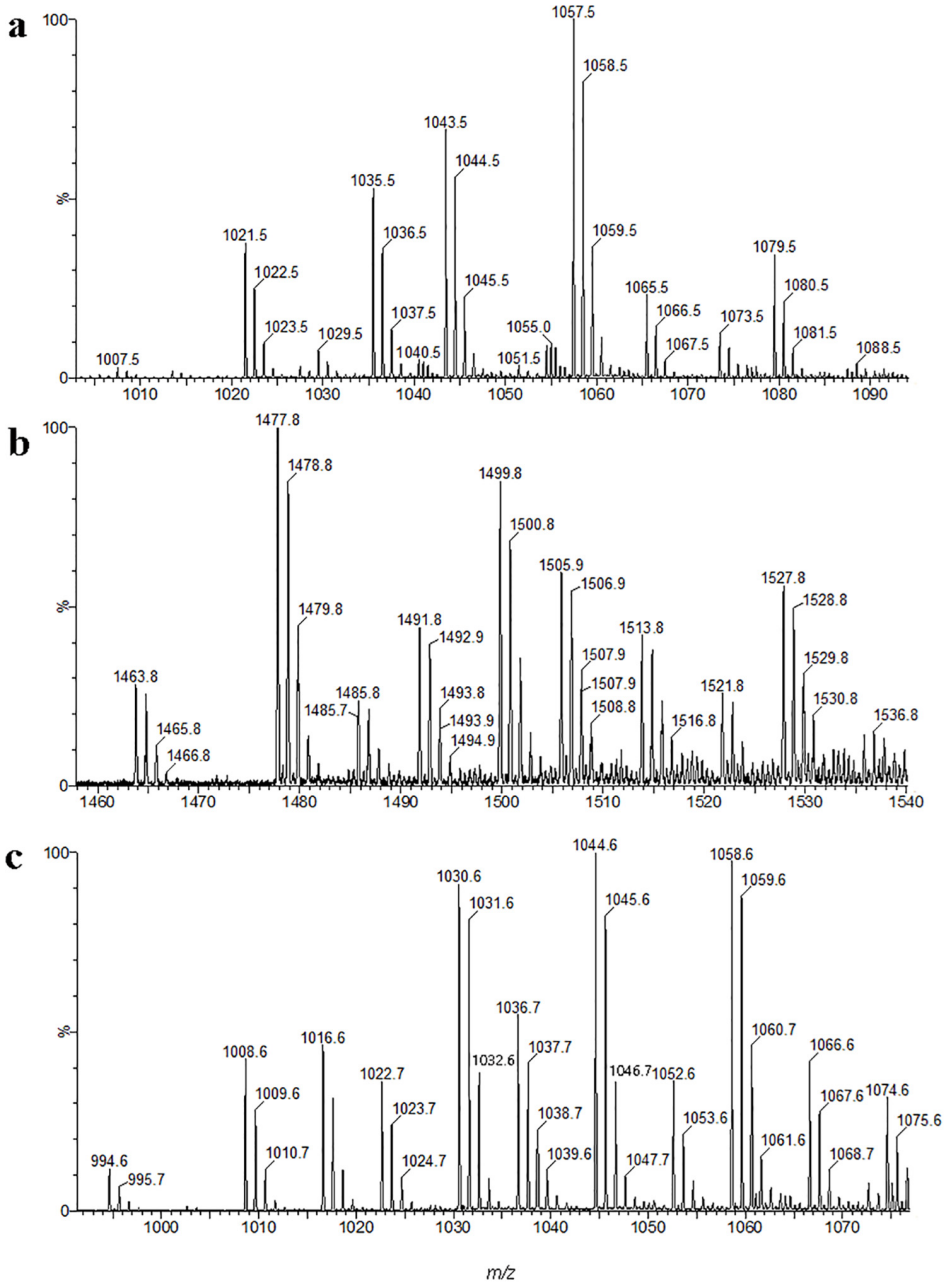
Mass spectra corresponding to an RP-HPLC chromatogram LC-MS of the methanolic fraction from antifungal substances. a: Mass spectra corresponding to the bacillomycin L homolog; b: Mass spectra corresponding to the fengycin homolog; c: Mass spectra corresponding to the surfactin homolog.

The results for the MALDI-TOF-MS spectra, which were listed in Table 3, indicated that there were three lipopeptide families with signals at m/z ranges from 1000 to 1100 (Fig 4a), 1400 to 1600 (Fig 4b), and 900 to 1100 (Fig 4c).

**Table 3:** Primary peaks detected by a UV-MALDI TOF MS analysis of the lipopeptides produced by strain SYBC H47.

Lipopeptide classes	Fatty acid chain	Calculated (*m/z*)			Fractions
		[M+H]^+^	[M+Na]^+^	[M+K]^+^	
Bacillomycin L					
	C13	1007.5	1029.5	1045.5	1–4
	C14	1021.5	1043.5	1059.5	
	C15	1035.5	1057.5	1073.5	
Fengycin A					
	C16	1463.8	1485.8	1501.8	5–8
	C17	1477.8	1499.8	1515.8	
Fengycin B					
	C16	1491.8	1513.8	1529.8	
	C17	1505.9	1527.8	1543.8	
Surfactin					
	C13	994.6	1016.6	1032.6	9–12
	C13,C14	1008.6	1030.6	1046.7	
	C14,C15	1022.7	1044.6	1060.7	
	C15	1036.7	1058.6	1074.6	

Fractions one to four consisted of a homolog of bacillomycin L. Three homologs with a heptapeptide moiety (L-Asp-D-Tyr-L-Asn-L-Ser-L-Gln-L-Ser-L-Thr) were detected as [M+H]^+^ at *m/z* = 1007.5, 1021.5, and 1035.5, corresponding to links with C13, C14, and C15 hydroxy fatty acid chains. The molecular weights of the sodium adducts [M+Na]^+^ were 1029.5, 1043.5, and 1057.5, corresponding to C13, C14, and C15. Furthermore, the potassium adduct [M+K]^+^ C13 at *m/z* was 1045.5, the C14 at *m/z* was 1059.5, and the C15 at *m/z* was 1073.5. The spectra were similar to those reported by Hourdou [45] and Aktuganov [46].

Fractions five to eight contained a homolog of fengycin. Two homologs of fengycin A with a decapeptide moiety (L-Glu-D-Orn-D-Tyr-D-Thr-L-Glu-D-Ala-L-Pro-L-Gln-L-Tyr-L-Ile) were detected as [M+H]^+^ at *m/z* = 1463.8 and 1477.8, corresponding to links with C16 and C17 hydroxy fatty acid chains. In addition, the molecular weights of sodium and potassium adducts [M+Na]^+^ and [M+K]^+^ for C16 were 1485.8 and 1501.8; and for C17 they were 1499.8 and 1515.8, respectively. With the difference of fengycin A, the fengycin B decapeptide moiety was L-Glu-D-Orn-D-Tyr-D-Thr-L-Glu-D-Val-L-Pro-L-Gln-L-Tyr-L-Ile. The molecular weights of the homologs were detected as [M+H]^+^ C16 at *m/z* = 1491.8 and C17 at *m/z* = 1505.9, with sodium and potassium adducts [M+Na]^+^ and [M+K]^+^ of C16 at *m/z* = 1513.8 and 1529.8; and C17 at *m/z* = 1527.8 and 1543.8, respectively. The spectra were similar to those reported by Williams [47] and Monaci [48].

Fractions nine to twelve contained a homolog of surfactin. The molecular weights of the surfactin homologs were detected as [M+H]^+^ at *m/z* = 1008.6, 1022.7 and 1036.7, corresponding to C13, C14, and C15 hydroxy fatty acid chains, which were linked to the heptapeptide moiety (L-Glu-L-Leu-D-Leu-L-Val-L-Asp-D-Leu-L-Leu). In addition, the sodium and potassium adducts [M+Na]^+^ and [M+K]^+^ were 1030.6 and 1046.7 for C13; 1044.6 and 1060.7 for C14; and 1058.6 and 1074.6 for C15. However, when the leucine was substituted by valine at position seven in heptapeptides, it was detected as [M+H]^+^ at *m/z* = 994.6, 1008.6 and 1022.7, for C13, C14, and C15, respectively. In addition, the sodium and potassium adducts [M+Na]^+^ and [M+K]^+^ were 1016.6 and 1032.6 for C13; 1030.6 and 1046.7 for C14; and 1044.6 and 1060.7 for C15. The spectra were similar to those reported by Yang [49] and Monaci [48].

### Effects of lipopeptides on the suppression of conidial germination

**Fig 5:**
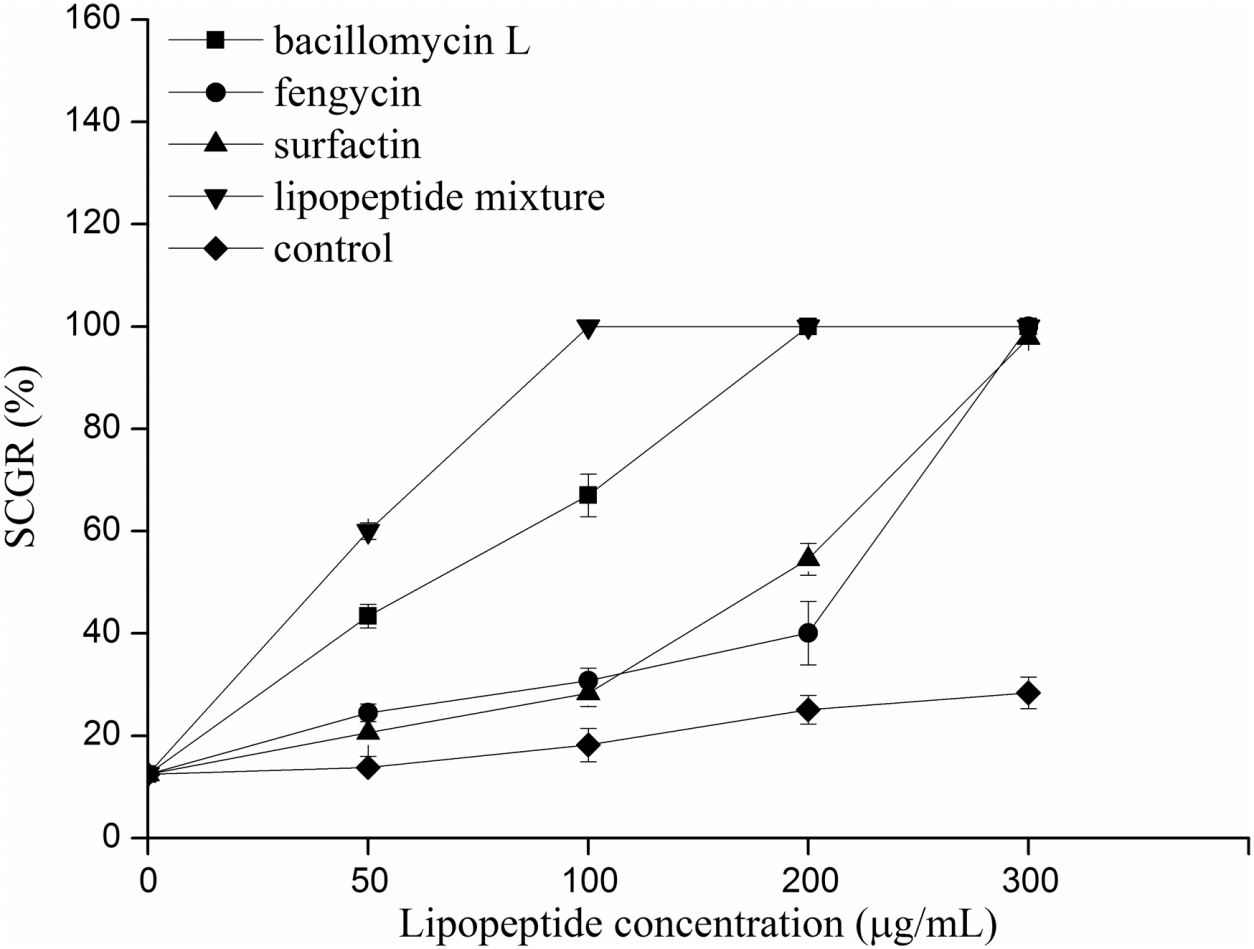
Effects of different lipopeptides on the suppression of conidial germination. The lipopeptide mixture was composed of bacillomycin: fengycin: surfactin (1:1:1, v/v/v).

The conidia of pathogenic fungi that were treated with lipopeptides revealed morphological modifications and a further loss of germination ability [50]. In this work, the conidial germination of *B*. *dothidea* that was treated with the lipopeptides produced by strain SYBC H47 was evaluated. The crude lipopeptides were separated and purified by TLC, redissolved in methanol, and then diluted with deionized water to different concentrations (0, 50, 100, 200, and 300 μg/mL). The methanol used as a control was diluted to the same concentration with deionized water. As revealed by the effect of different lipopeptides on the suppression of conidial germination (Fig 5), the lipopeptide mixture showed the greatest intensity of antifungal activity against *B*. *dothidea* conidial germination, and its SCGR reached 100% by concentration at 100 μg/mL. Bacillomycin L also exhibited intense antifungal activity; its SCGR reached above 40% at 50 μg/mL, 60% at 100 μg/mL, and 100% at 200 μg/mL. Moreover, both fengycin and surfactin had similar antifungal activities against conidia. At a low concentration (below 100 μg/mL), the SCGR levels were both below 30%. When the concentration increased (200–300 μg/mL), the SCGR levels markedly increased.

### Effects of lipopeptides on the suppression of mycelial growth

**Fig 6:**
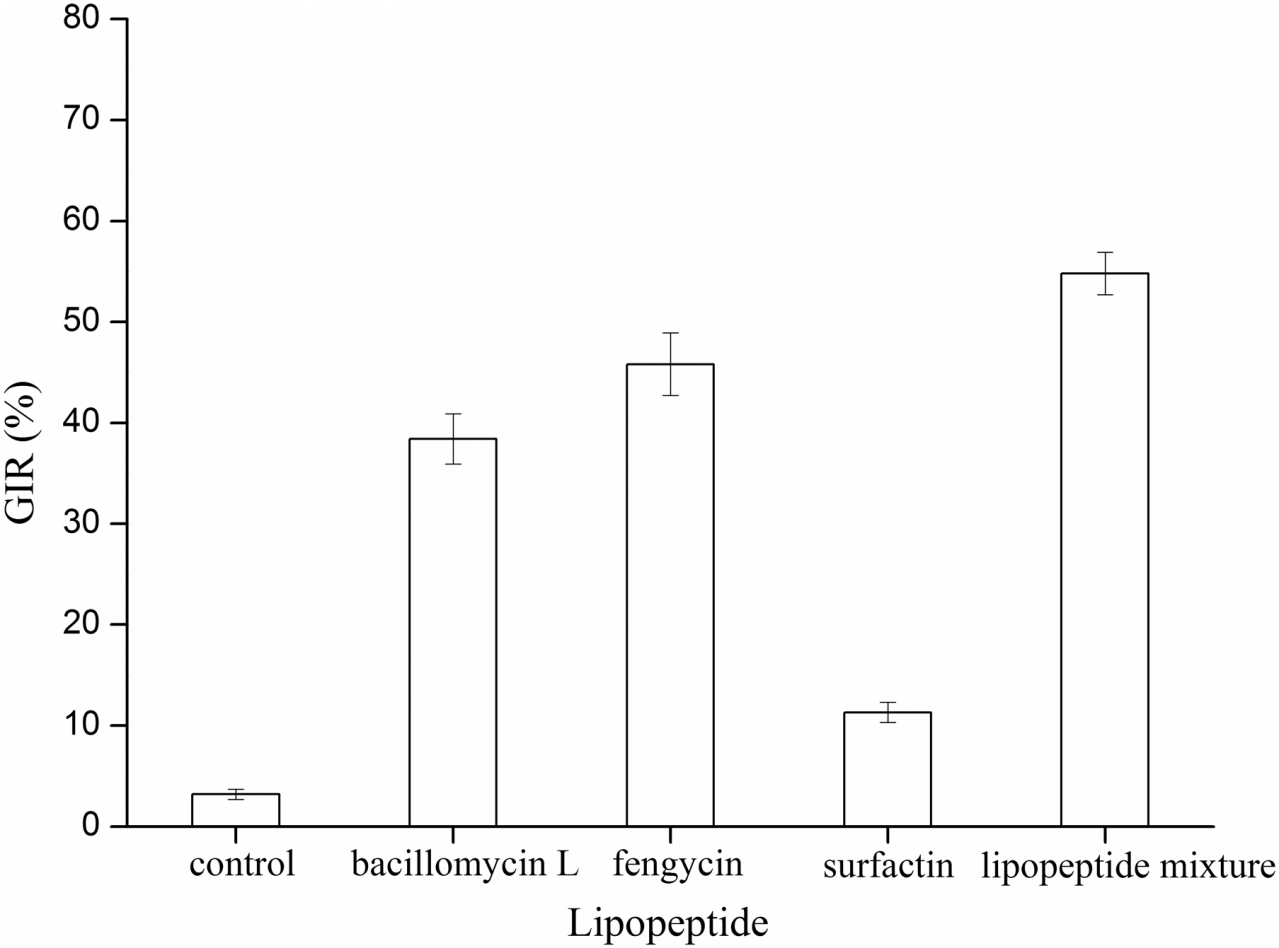
Effects of different lipopeptides on the suppression of mycelial growth. The lipopeptide concentration was 100 μm/mL.

A further exploration of the properties of antifungal substances involved in mycelial growth inhibition were obtained by performing agar diffusion assays *in vitro*. The bacillomycin, fengycin, surfactin, and lipopeptide mixture at 100 μm/mL were investigated against the mycelial growth of *B*. *dothidea*. As Fig 6 shows, surfactin had a limited effect in terms of inhibiting the growth of *B*. *dothidea*. In comparison with surfactin, bacillomycin L and fengycin had similarly intense antifungal activities to suppress the growth of *B*. *dothidea*, with GIR values between 30 to 40%. In contrast to the separated lipopeptides, the lipopeptide mixtures exhibited the maximum GIR (approximately 50%).

### Effects of lipopeptide mixture on cell membrane permeability

**Fig 7:**
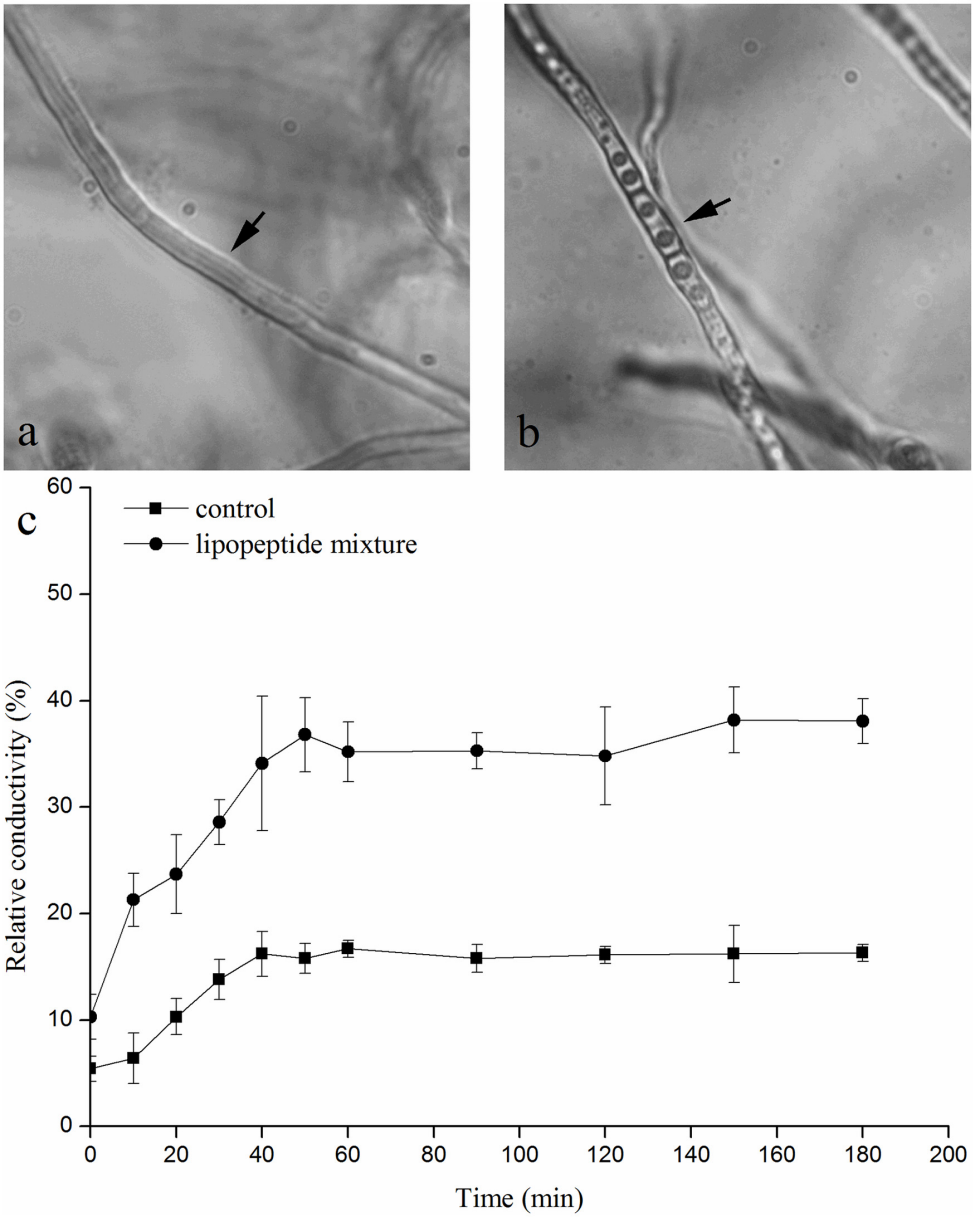
Effects of the lipopeptide mixture and water on the cell membranes of mycelia. The concentration of the lipopeptide mixture was 100 μm/mL. a: Mycelia treated with water; b: mycelia treated with lipopeptide mixture; c: relative conductivity of *B*. *dothidea* mycelia grown in PDB amended with 100 μm/mL of lipopeptide mixture. Black arrow: mycelia of strain SYBC H47.

The mycelia of *B*. *dothidea* were treated with a lipopeptide mixture (100 μm/mL) and compared with a water control. In comparison with the untreated mycelia (Fig 7a), morphological abnormalities of mycelia were induced by lipopeptide mixture liquor. The large vacuole appeared, cytoplasm condensed and septa easily observed in the swollen mycelial cells (Fig 7b). According to the cell membrane permeability variation (Fig 7c), which was represented by the relative conductivity, the results showed that the lipopeptide mixture liquor significantly increased the cell membrane permeability of the mycelia (by approximately 20%) over 60 min.

### Low-cost carbon source and lipopeptide production medium

To reduce costs, waste frying oils were evaluated for lipopeptide production by strain SYBC H47 through batch fermentation. As shown in Table 4, the use of waste frying peanut oil and waste frying soy oil increased the level of lipopeptide production as the sole carbon source by approximately 17% (2.42 g/L) and 110% (4.35 g/L) in comparison with the use of Landy medium, respectively. These results are consistent with those of Haba *et al* [51]. The results showed that the proportions of three lipopeptides were influenced by different carbon sources. The use of waste frying soy oil was beneficial for synthesizing bacillomycin L (approximately 6%) and fengycin (approximately 3%), relative to the use of the control, and reduced the surfactin level by approximately 8%. The use of waste frying peanut oil increased fengycin to its highest level (42.79%). Considering the economic advantage of using this inexpensive waste oil as the carbon source for lipopeptide production, waste frying oil was selected for further study.

**Table 4:** Lipopeptide concentration produced by strain SYBC H47 when using different oil substrates as the sole carbon source.

Substrates	Lipopeptide concentration (g/L)	Bacillomycin L (%)	Fengycin (%)	Surfactin (%)
waste frying peanut oil	2.42 ± 0.41^b^	30.35	42.79	26.86
waste frying soy oil	4.35 ± 0.26^a^	42.22	37.55	20.23
Landy medium (control)	2.07 ± 0.17^b^	36.70	34.76	28.54

The *different letters* (a and b) listed after each number represent significant difference between treatments and control (*P*<0.05) by Duncan’s test.

### Antifungal activity in the field

The SYBC H47 strain was further tested for its potential to reduce peach gummosis disease in local orchards. As shown in Table 5, although none of the treatments repaired the bark scars and completely inhibited the gummosis disease, the cell suspension and lipopeptide liquor could significant reduce the IGR and DSI. The control effect of the cell suspension was superior to that of lipopeptide liquor. In 2014, when treating with cell suspension and lipopeptide liquor, the IGR decreased by 20% and 10%, and the DSI decreased by 57.5% compared with the control. Furthermore, the continuous application of cell suspension and lipopeptide liquor led to a better control effect. The IGR in the cell suspension treatment was 60%, and the DSI was 22.5%. However, the carbendazol had limited effectiveness in controlling gummosis disease because this compound was applied to the orchard for several years. Hence, the metabolites synthesized by the SYBC H47 strain had potential use as an alternative biocontrol agent against gummosis disease.

**Table 5:** Effects of the disease incidence rate and disease severity index of gummosis for different treatments in the field trial.

Treatment	Cell suspension	Lipopeptide liquor	Carbendazol	Water
2014				
IGR (%)	80.0^b^	90.0^ab^	100.0^a^	100.0^a^
DSI (%)	27.5^c^	32.5^c^	57.5^b^	85.0^a^
2015				
IGR (%)	60.0^c^	80.0^b^	100.0^a^	100.0^a^
DSI (%)	22.5^c^	30.0^c^	60.0^b^	80.0^a^

For the data for treatments that were summarized from the same trees in 2014 and 2015, each treatment had 10 replications. The suspension concentration was 2×10^6^ cfu/mL and the carbendazol concentration was 200 μg/mL. Distilled water was used as a control. The *different letters* (a, b and c) listed after each number represent significant difference between treatments and control (*P*<0.05) by Duncan’s test.

## Discussion

Honey is a desirable food source that has attracted more attention because of the production of antifungal compounds from its bacterial isolates [24, 52]. In this study, 225 bacterial strains were isolated from raw honey, strain SYBC H47 had the strongest antifungal activity against *B*. *dothidea*. According to its physiological and biochemical characteristics, API 50 CHB examinations, 16S rRNA gene and *gyrB* gene analysis (Fig 1), the SYBC H47 strain was identified as *B*. *amyloliquefaciens*. This is the first report on the use of *B*. *amyloliquefaciens* isolated from honey for the biocontrol of peach gummosis. An interesting finding was that the ampicillin tolerance of *B*. *amyloliquefaciens* SYBC H47 was up to 200 μg/mL.

Many reports have indicated that several antifungal mechanisms of *Bacillus* species contribute to phytopathogen antagonism. For example, siderophores, which are important natural iron chelators, represent a novel class of antibiotics with considerable therapeutic potential [53]. Chitinase acts as an antagonist against fungi by inhibiting spore germination and germ-tube elongation [32]. β-1,3-glucanase is able to lyse the cell walls of *F*. *oxysporum*, *Rhizoctonia solani*, *Sclerotium rolfsii* and *Pythium ultimum* [54]. Protease helps to inhibit the growth of fungi by hydrolyzing the cell wall [55]. Furthermore, lipopeptides that are produced by *B*. *amyloliquefaciens* play an important role in controlling plant diseases. It was interesting that the antifungal activities of a cell suspension produced by *B*. *amyloliquefaciens* SYBC H47 were much higher than that of its cell-free supernatant (Fig 2). The appropriate explanations for this phenomenon may be attributed to the following reasons. *B*. *amyloliquefaciens* produces different antifungal substances when it is cultured in heterogeneous medium [56]. In addition, when interacting with fungi, the antifungal mechanisms of *B*. *amyloliquefaciens* can be fine-tuned by producing different antifungal substances. In this study, the SYBC H47 strain could synthesize several antifungal enzymes, such as chitinase, β-1,3-glucanase [57], and protease [46]. These antifungal enzymes, which are expressed by *Bacillus* cells upon interaction with corresponding inducers, belong to the induced enzymes. However, the antifungal enzymes were deficient in the cell-free supernatant of strain SYBC H47, and hence the antifungal activity was lower compared with that of the cell suspension. Moreover, the SYBC H47 strain that was cultured in submerged liquid fermentation or solid medium could produce different amounts of antifungal substances, except for antifungal enzymes, and this result was consistent with that of report [58].

The LC/ESI-MS/MS analysis (Fig 4) of culture filtrates indicated that there were three different types of lipopeptides produced by *B*. *amyloliquefaciens* SYBC H47 simultaneously, namely bacillomycin L, fengycin and surfactin (Table 3). The antifungal activity results against *B*. *dothidea* showed that the conidia were sensitive to lipopeptides in a dose-dependent manner (Fig 5). Bacillomycin L was the most effective lipopeptide to inhibit conidial germination, and it had a similar effect on the mycelia. Bacillomycin L reportedly acts with strict sterol-phospholipid dependence on the membrane formation ternary structure, iturin/phospholipid/sterol [59, 60]. Nevertheless, research by Zhang [61] showed that bacillomycin L was not restricted to interactions with sterol. In addition, fengycin was able to effectively suppress the growth of mycelia. Similar observations are also reported, and they showed that fengycin interferes with the tight packing of membrane phospholipids. Fengycin aggregates to form pores, causing permeability changes in the membrane at low concentrations, whereas it behaves as a detergent by solubilizing the membrane at a high concentration [62]. However, surfactin had a similar suppressive effect on conidial germination as that of fengycin, and it had limited action on the growth of mycelia (Fig 6). This result was slightly different from that of previous research [52]. It is well-know that surfactin is an acidic lipopeptide, and it can destabilize of lipid-packing of membranes and induce content leakage, but the membrane-perturbing effect is attenuated by adding sterol [63]. Our present study showed that the lipopeptide mixture was most effective at suppressing both the germination of conidia and the growth of mycelia because it had additive or synergistic effects on conidial germination suppression and cell membrane permeability (Fig 7). These results were consistent with those reported in [64].

Lipopeptides offer remarkable applications to diverse fields; however, their high production cost is the primary obstacle to their commercial application. To reduce fermentation costs, many researchers have used low-cost raw substrates to produce lipopeptides, such as rice straw [65] and potato [66] and orange peels [67]. Large quantities of waste frying oil are generated by the food industry. In using these oils for a long time at high temperatures, harmful compounds that impact human health are produced in oil. However, these waste frying oils that contain high-energy hydrocarbons can be recycled, and as a result, they can be used as a low-cost carbon source to produce lipopeptides.

A great deal of researches shows that the higher antifungal activity of lipopeptides is influenced by the higher ratio of iturin and fengycin in lipopeptides. The composition ratio of lipopeptides could be changed in different media (Table 4). The glucose in Landy medium was more suitable for bacterial cell growth, and it led to a shortened cell logarithmic growth phase. At the same time, surfactin synthesis was accompanied by the cell logarithmic growth phase, and bacillomycin L and fengycin were synthesized at the late logarithmic phase as well as the stationary phase. When waste frying soy oil, rich oleic acid and linoleic acid, were used as sole carbon sources, they could lower the growth rate and offer precursors for lipopeptide synthesis.

In the field trial (Table 5), the suspension and cell-free supernatant of *B*. *amyloliquefaciens* SYBC H47 could effectively reduce the disease incidence rate and the disease severity index of gummosis, with the exception of carbendazol. Carbendazol may have been used over the long term to control gummosis in peach orchards, leading to resistance in the *Botryosphaeria* species [6]. Damaged peach tissue caused by gummosis provides an entry point for pathogen invasion, whereas *B*. *amyloliquefaciens* SYBC H47 could not repair bark scars. Thus, routinely spraying a suspension will be an acceptable strategy for suppressing gummosis breakouts.

In conclusion, *B*. *amyloliquefaciens* SYBC H47 that was isolated from honey was a worthy biocontrol agent against *B*. *dothidea in vitro* and inhibited gummosis in a field trial. In exploring the mechanisms of the antagonist, *B*. *amyloliquefaciens* SYBC H47 was found to produce bacillomycin L, fengycin, surfactin, chitinase, β-1,3-glucanase, cellulose, siderophores and IAA. Waste soy frying oil could be used as the sole carbon source to increase lipopeptide production. All these products would play a role in simultaneously offering better and more efficient disease control. In addition, a few antifungal enzymes and compounds produced by *B*. *amyloliquefaciens* SYBC H47 were correlated with plant growth promotion. Thus, *Bacillus* spp. isolated from honey is a novel potential resource to explore for use as biocontrol agents against plant disease.
